# Effectiveness of Transcranial Magnetic Stimulation on Upper Limb Fine Motor Skills in Post-stroke Patients: A Systematic Review and Meta-Analysis

**DOI:** 10.7759/cureus.98280

**Published:** 2025-12-01

**Authors:** Mothana Al Jaber, Khalid M Alsulaim, Riad A Kouzeiha, Abdulaziz A Alsuhaibani, Razan A Lasloom, Yara Alghamdi, Khadijah Basheikh, Saud Alasmari, Rahma Saad, Hazem Abdelkhalek

**Affiliations:** 1 Department of Neurology, College of Medicine, King Faisal University, Al Ahsa, SAU; 2 College of Medicine, Qassim University, Unaizah, SAU; 3 Department of Medicine, Lebanese University, Hadath Campus, Beirut, LBN; 4 Department of Medicine, Qassim University, Al-Qassim, SAU; 5 College of Medicine, Najran University, Najran, SAU; 6 Department of Medicine and Surgery, Faculty of Medicine, King Abdulaziz University, Jeddah, SAU; 7 Faculty of Medicine, ‏King Abdulaziz University, Jeddah, SAU; 8 King Khalid University, College of Medicine, Abha, SAU; 9 Faculty of Medicine, Modern University for Technology and Information (MTI), Cairo, EGY; 10 Department of Neurology/Interventional Neurology, Tanta University, Tanta, EGY

**Keywords:** dexterity, fine motor skills, grip strength, meta-analysis, pinch strength, randomized controlled trials, stroke rehabilitation, transcranial magnetic stimulation

## Abstract

Stroke frequently results in persistent upper limb fine motor deficits, severely impairing daily activities. While transcranial magnetic stimulation (TMS) is increasingly applied as an adjunct therapy, its effectiveness in restoring dexterity-specific outcomes remains unclear. This systematic review and meta-analysis evaluated the effectiveness of TMS in improving fine motor skills among post-stroke patients. Following PRISMA (Preferred Reporting Items for Systematic Reviews and Meta-Analyses) guidelines, five databases (PubMed, Embase, Web of Science, Cochrane Library, Google Scholar) were searched up to March 2025. Only randomized controlled trials (RCTs) reporting upper limb fine motor outcomes in post-stroke patients undergoing TMS were included. Data were extracted and analyzed using RevMan 5.4.1, with mean differences (MDs) and 95% confidence intervals (CIs) calculated under a random-effects model. Risk of bias was assessed using the Cochrane Risk of Bias-2 (ROB-2) tool. Of 1,416 screened studies, eight RCTs met eligibility, including 257 participants (127 rTMS vs. 130 controls). Study follow-up ranged from one to two months. Four studies were rated high quality, and four showed moderate concerns. Pooled analysis demonstrated no significant improvements with rTMS over control in the Box and Block test (MD: 0.75; 95% CI -4.62 to 6.11; *P *= 0.79), grip strength (MD: 2.49; 95% CI -0.18 to 5.16; *P *= 0.07), or pinch strength (MD: 0.65; 95% CI -0.39 to 1.69; *P *= 0.22). Subgroup analyses and sensitivity tests confirmed these findings. Current evidence does not support a significant benefit of TMS for fine motor recovery post-stroke, despite its established role in gross motor rehabilitation. Larger, high-quality RCTs targeting dexterity-specific outcomes, stimulation parameters, and patient stratification are needed to clarify TMS’s potential in fine motor rehabilitation.

## Introduction and background

Stroke remains one of the foremost causes of long-term disability worldwide, with upper limb motor impairments affecting approximately 50%-80% of survivors [1]. Among these impairments, deficits in fine motor skills, such as dexterity, precision, and coordination of hand and finger movements, pose a significant barrier to independence in activities of daily living (ADLs), including writing, fastening clothing, and manipulating small objects [2]. Although nearly two-thirds of survivors regain ambulatory ability, fewer than half achieve meaningful restoration of fine motor control within the first year after stroke [3]. This disparity underscores the urgent need for innovative interventions that harness neuroplasticity to facilitate recovery of intricate hand functions critical for daily independence.

Traditional rehabilitation methods often produce only modest improvements in fine motor skills after chronic stroke [4]. This limited effect is largely due to stroke-related damage within the motor network, including M1, premotor areas, and corticospinal pathways that are essential for precise hand control [5]. Non-invasive brain stimulation, particularly transcranial magnetic stimulation (TMS), has emerged as a useful addition to standard therapy by adjusting cortical activity and restoring balance between the hemispheres [6]. Repetitive TMS, whether applied as high-frequency stimulation to the affected side or low-frequency stimulation to the opposite hemisphere, has shown promise in enhancing neuroplasticity and supporting motor recovery [3,7].

Previous meta-analyses largely support the use of TMS for enhancing upper limb motor outcomes. For example, Li et al. found that both excitatory and inhibitory rTMS significantly improved Fugl-Meyer Assessment Upper Extremity (FMA-UE) scores, with outcomes varying by stroke stage and severity of hemiplegia [3]. Similarly, Zhang et al. reported lasting improvements in dexterity and grip strength, particularly when TMS was delivered during the acute phase or with intermittent theta-burst stimulation (iTBS) [7]. However, these studies have predominantly emphasized composite measures (e.g., FMA-UE, Barthel Index) or gross motor recovery, with comparatively limited attention to fine motor outcomes such as precision grip, finger individuation, and functional hand tasks [3,8]. Fine motor deficits are especially detrimental to quality of life and require distinct neural mechanisms and rehabilitation strategies compared to proximal limb recovery [9]. For instance, finger dexterity is critically dependent on corticospinal tract integrity and M1 reorganization, which may respond differently to TMS protocols (e.g., frequency, stimulation site) compared to proximal motor functions of the shoulder or elbow [10].

Despite encouraging evidence, several critical gaps remain. First, earlier systematic reviews often merged fine and gross motor outcomes, obscuring the specific contribution of TMS to dexterity and precision [6]. Second, the role of timing after stroke, lesion location (cortical versus subcortical), and baseline severity of impairment in shaping fine motor outcomes is not yet fully elucidated [7]. Addressing these limitations is essential for optimizing TMS protocols and tailoring rehabilitation to maximize functional recovery.

A systematic review and meta-analysis are therefore warranted to consolidate the fragmented and inconsistent evidence regarding the efficacy of TMS for fine motor recovery after stroke. Although individual trials have explored this relationship, most are constrained by small sample sizes, heterogeneous methodologies, and a predominant focus on broad motor outcomes rather than dexterity-specific measures [7]. This review aims to provide a clear and comprehensive evaluation of how effective TMS is in improving fine motor function after stroke.

## Review

Methodology

The PRISMA (Preferred Reporting Items for Systematic Reviews and Meta-Analyses) guidelines were followed throughout this systematic review and meta-analysis [11]. The protocol of the review is registered in PROSPERO under the ID (CRD42024624035).

Search strategy

We searched PubMed, Google Scholar, Embase, Web of Science (WOS), and the Cochrane Library from inception to March 2025 to identify studies examining the use of TMS for improving upper limb fine motor skills in individuals following stroke. The search terms: ("Stroke" OR "Cerebrovascular Accident") AND ("Transcranial Magnetic Stimulation" OR "TMS") AND ("Fine Motor Skills" OR "Dexterity") "Post-Stroke" AND "rTMS" AND "Upper Limb" AND ("Standard Rehabilitation" OR "Sham TMS"). Additionally, we screened the reference lists of all included studies to identify any relevant articles that may have been missed during the database search.

Inclusion and exclusion criteria

The inclusion criteria for the review were as follows: studies reporting upper limb fine motor skill tests only, randomized controlled studies only, and patients who had a stroke and were receiving TMS. Exclusion criteria included studies that involved patients receiving other treatment modalities; studies that did not report upper limb fine motor skills; and editorials, case reports, posters, conference proceedings, unpublished articles, books, and reviews.

Data extraction

Two reviewers independently extracted data using a predefined, standardized form. The collected information included study details (first author, publication year, country, design, sample size, and follow-up period), participant characteristics (age and sex distribution), and outcome measures (Box and Block test, grip strength, and pinch strength). Any differences in the extracted data were addressed through discussion and resolved by agreement.

Quality assessment

We used the Cochrane Risk of Bias (ROB) assessment tool, as outlined in the Cochrane Handbook for Systematic Reviews of Interventions, version 6.0. This tool identifies five types of bias: performance bias, selection bias, detection bias, reporting bias, and attrition bias. Based on these domains, each included RCT was categorized as having a high, unclear, or low ROB.

Data analysis

Data analysis was conducted using Review Manager (RevMan) version 5.4.1. The extracted trial data were evaluated using mean difference (MD) and corresponding standard deviations, applying a random-effects model with the inverse variance statistical method. Results were reported as MDs with 95% confidence intervals (CI) and *P*-values, with statistical significance defined as *P* < 0.05. Heterogeneity was assessed using the I² statistic. Subgroup analyses were performed to evaluate the efficacy of treatment before and after the intervention. The calculation of changes between pre- and post-treatment values was conducted using a specially designed calculation sheet. To ensure consistency, these calculations were independently performed by two authors.

Results

**Figure 1:**
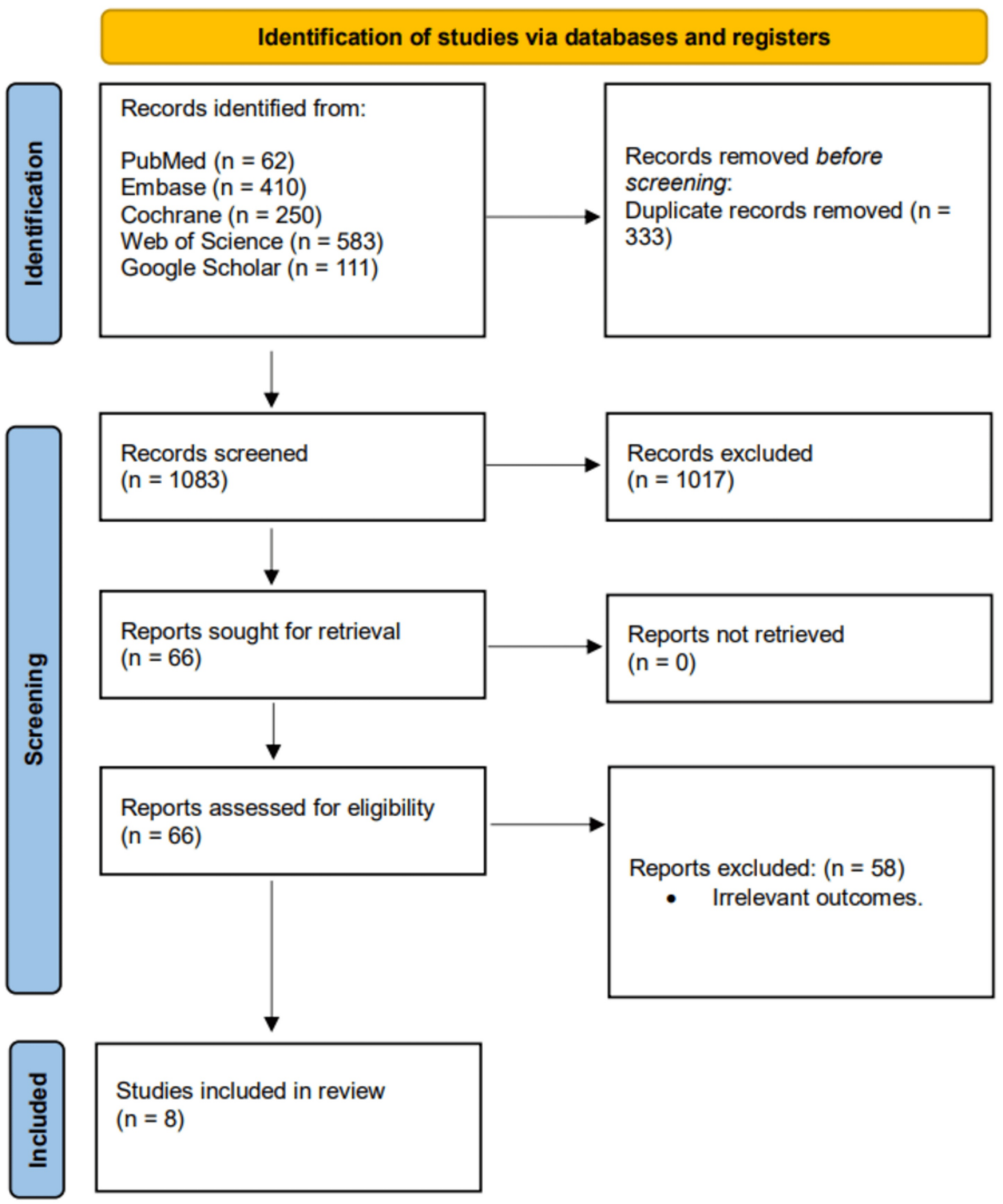
PRISMA flowchart. PRISMA, Preferred Reporting Items for Systematic Reviews and Meta-Analyses

An extensive search of PubMed, WOS, Google Scholar, Embase, and the Cochrane Library initially yielded 1,416 studies. After removing duplicates, 1,083 unique records remained and were screened by title and abstract, resulting in the exclusion of 1,017 studies. The remaining 66 full-text articles were independently assessed for eligibility, of which 8 studies met the inclusion criteria and were included in the final analysis. The study selection process is illustrated in the PRISMA flow diagram (Figure 1).

Characteristics of the included studies

A total of 8 studies were included in this systematic review and meta-analysis [12-19]. Among them, five studies were conducted in South Korea, one in China, one in Iran, and one in Turkey. Regarding the total number of participants, Group A included 127 patients (receiving repetitive TMS (rTMS)), while Group B comprised 130 patients (receiving sham stimulation or standard rehabilitation). The follow-up periods varied across studies, ranging from one month to two months, with most studies not reporting follow-up duration. The mean ages of participants in both groups ranged between 50 and 71 years, reflecting a primarily middle-aged and elderly cohort. Full details about the characteristics of the included studies are available in Table 1.

**Table 1:** Characteristics of the included studies. RCT, randomized controlled trial; rTMS, repetitive transcranial magnetic stimulation; ML, motor learning; HF-rTMS, high-frequency repetitive transcranial magnetic stimulation; TOMT, task-oriented motor training

Study	Country	Design	Participants		Mean age		Male		Female		Follow-up
			Group A	Group B	Group A	Group B	Group A	Group B	Group A	Group B	
Yang et al., 2021 [13]	China	RCT	rTMS 14	Hand grip training alone; 13	rTMS 61 ± 10	Hand grip training alone; 64 ± 8	rTMS 10	Hand grip training alone; 4	rTMS 8	Hand grip training alone; 5	NA
Shim et al., 2023 [14]	South Korea	RCT	HF-rTMS + ML 17	Sham rTMS + ML 16	HF-rTMS + ML 67.28 ± 10.80	Sham rTMS + ML 63.56 ± 16.09	HF-rTMS + ML 7	Sham rTMS + ML 11	HF-rTMS + ML 7	Sham rTMS + ML 5	NA
Haghighi et al., 2021 [15]	Iran	RCT	HF-rTMS Group 10	Control Group (n=10)	HF-rTMS Group 50.50 ± 9.47	Control Group 53.90 ± 13.06	HF-rTMS Group 6	Control Group 5	HF-rTMS Group 4	Control Group 5	NA
Lee et al., 2024 [16]	South Korea	RCT	Cr-Cbll Group (n = 10)	Cr-Sham Group (n = 10)	Cr-Cbll Group 71.6 ± 12.0	Cr-Sham Group 66.7 ± 9.8	Cr-Cbll Group 7	Cr-Sham Group 5	Cr-Cbll Group 3	Cr-Sham Group 5	2 months
Kim et al., 2020 [17]	South Korea	RCT	Real rTMS (n = 36)	Sham rTMS (n = 37)	Real rTMS 61.2 (11.2)	Sham rTMS 62.9 (13.1)	Real rTMS 21	Sham rTMS 24	Real rTMS 15	Sham rTMS 13	1-month follow-up,
Kim et al., 2018 [18]	South Korea	RCT	HF-rTMS + TOMT 8	HF-rTMS 12	HF-rTMS + TOMT 51 ± 2.98	HF-rTMS 74.11 ± 2.88	HF-rTMS + TOMT 4	HF-rTMS 4	HF-rTMS + TOMT 4	HF-rTMS 8	NA
Ji et al., 2014 [19]	South Korea	RCT	Mirror plus rTMS group (n = 12)	Control group (n = 12)	Mirror plus rTMS group 54.73 ± 7.88	Control group 52.45 ± 8.08	Mirror plus rTMS group 7	Control group 8	Mirror plus rTMS group 5	Control group 4	NA
Aşkın et al., 2017 [20]	Turkey	RCT	TMS group 20	Control group 20	TMS group 56.75 ± 11.46	Control group 58.80 ± 12.02	TMS group 14	Control group 15	TMS group 6	Control group 5	NA

Quality assessment

**Figure 2:**
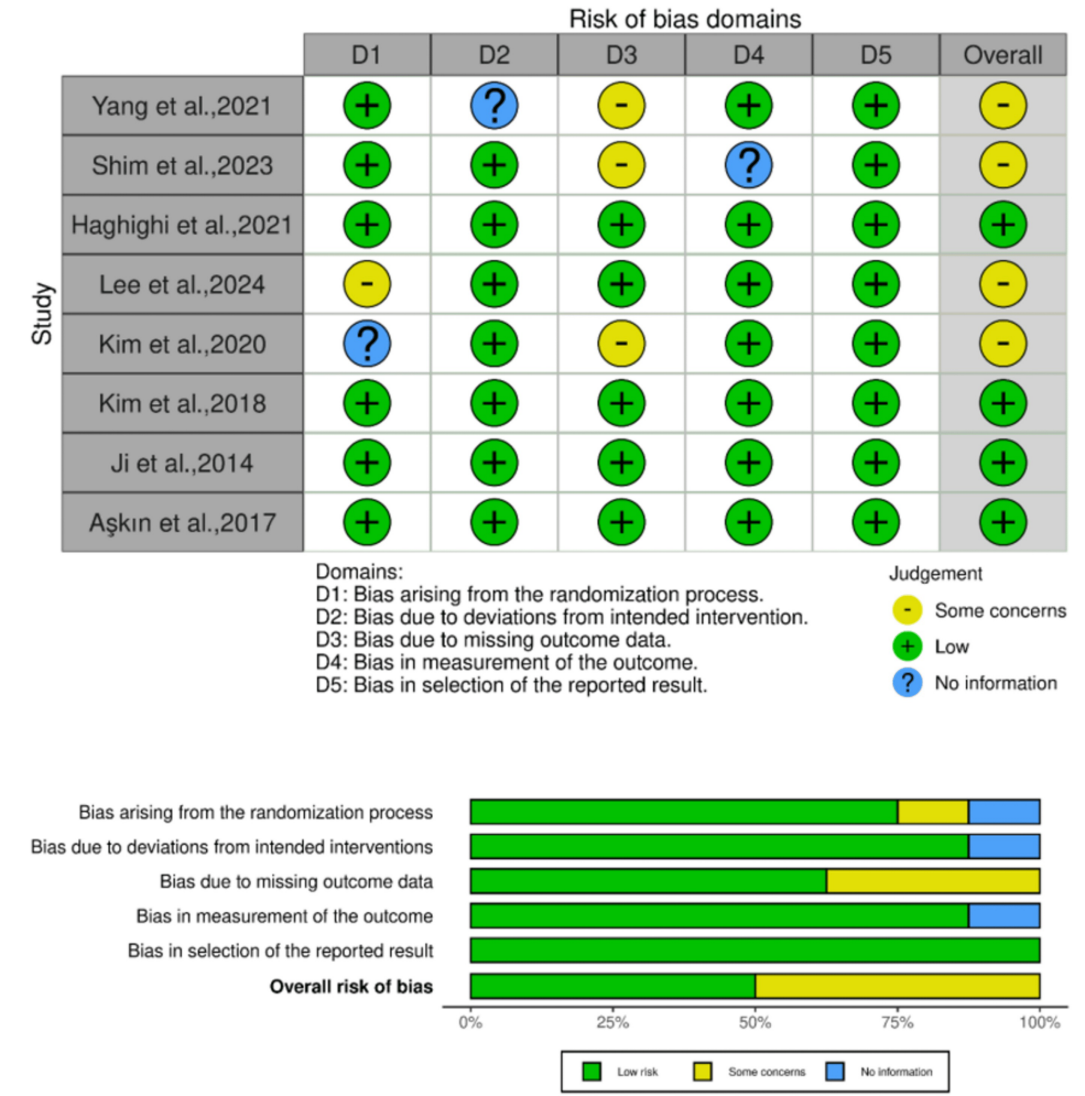
Quality assessment of the included studies using ROB-2 tool. [13-20]. ROB, Risk of Bias

The quality of the included studies was assessed using the ROB-2 tool. Four studies were considered to have some concerns, indicating moderate quality. While four studies were considered to have a low ROB, indicating high quality (Figure 2).

Outcomes

Box and Block test

**Figure 3:**
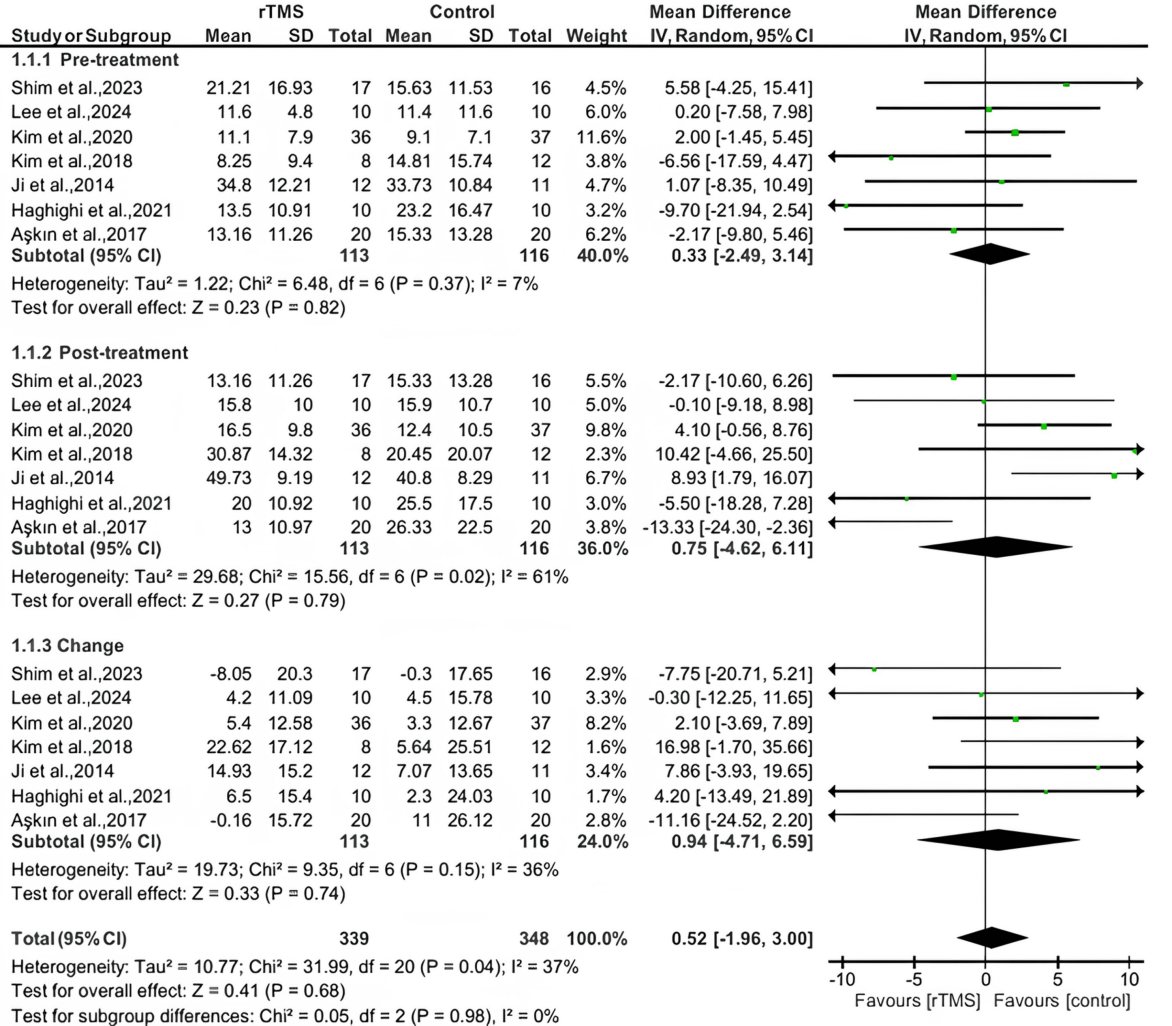
Pooled meta-analysis for the Box and Block test. [14,16-20]. rTMS, repetitive transcranial magnetic stimulation; CI, confidence interval; SD, standard deviation

Seven studies reported the Box and Block test outcome. Our analysis revealed no significant difference between the rTMS and control groups regarding the box and block test before and after the treatment, with mean differences (MD: 0.33, 95% CI: -2.49 to 3.14, *P*-value = 0.82, *I*^2^ = 7%), and (MD: 0.75, 95% CI: -4.62 to 6.11, *P*-value = 0.79, *I*^2^ = 61%), respectively. A meta-analysis was performed to resolve the high heterogeneity post-treatment. When the study by Aşkın et al. [20] was removed, the heterogeneity reduced to 32%. Also, there was no difference between the groups in the change from baseline to the end of follow-up (MD: 0.94, 95% CI: -4.71 to 6.59, *P*-value = 0.74, *I*^2^ = 36%) (Figure 3).

Grip strength

**Figure 4:**
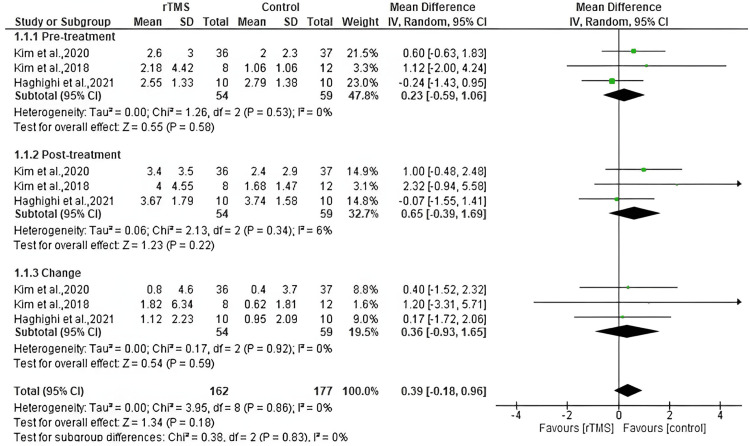
Pooled meta-analysis for grip strength. [13,15,17,18]. rTMS, repetitive transcranial magnetic stimulation; CI, confidence interval; SD, standard deviation

Four studies reported the grip strength outcome. Our analysis revealed no significant difference between the rTMS and control group regarding grip strength before and after the treatment, with mean differences (MD: 0.90, 95% CI: -1.07 to 2.86, *P*-value = 0.37, *I*^2^ = 0%), and (MD: 2.49, 95% CI: -0.18 to 5.16, *P*-value = 0.07, *I*^2^ = 0%), respectively. Also, no difference between the groups in change from baseline to the end of follow-up (MD: 2.05, 95% CI: -1.71 to 5.81, *P*-value = 0.29, *I*^2^ = 0%) (Figure 4).

Pinch strength

**Figure 5:**
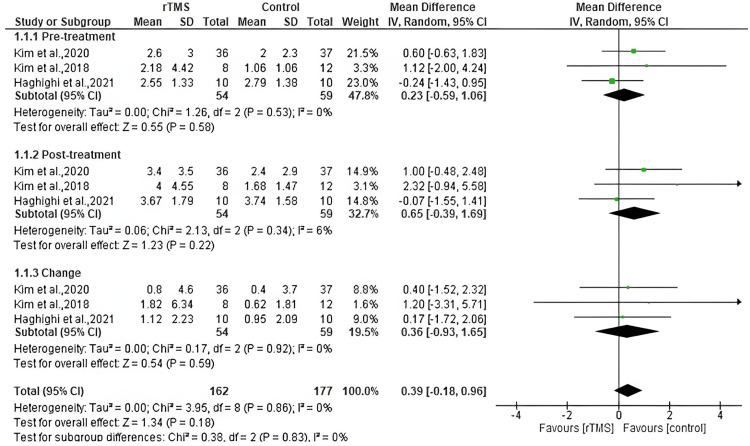
Pooled meta-analysis for pinch strength. [15,17,18]. rTMS, repetitive transcranial magnetic stimulation; CI, confidence interval; SD, standard deviation

Three studies reported the pinch strength outcome. Our analysis revealed no significant difference between the rTMS and control group regarding pinch strength before and after the treatment, with mean differences (MD: 0.23, 95% CI: -0.59 to 1.06, *P*-value = 0.58, *I*^2^ = 0%), and (MD: 0.65, 95% CI: -0.39 to 1.69, *P*-value = 0.22, *I*^2^ = 6%), respectively. There was no significant difference between the groups in the change from baseline to the end of follow-up (MD: 0.36, 95% CI: -0.93 to 1.65, *P*-value = 0.59, *I*^2^ = 0%) (Figure 5).

Discussion

The primary aim of this review was to evaluate the effectiveness of TMS in improving fine motor skills, namely dexterity, grip strength, and pinch strength, in post-stroke patients. Despite the growing body of evidence supporting TMS as a valuable adjunct in promoting upper limb recovery, our analysis found no significant improvements in fine motor outcomes across the included randomized controlled trials. This finding contrasts with the consistent benefits reported for gross motor function, where TMS has demonstrated measurable gains on composite scales such as the Fugl-Meyer Assessment. The lack of effect on dexterity is particularly important because fine motor control underpins essential daily activities, writing, buttoning clothing, or manipulating small objects, yet it remains one of the least recovered domains following stroke. These results highlight a critical gap in current neuromodulation strategies. While TMS appears effective for proximal motor restoration, its capacity to facilitate the recovery of precise, intricate hand movements remains limited and underexplored.

Our meta-analysis of seven trials employing the Box and Block Test (BBT) revealed no significant benefit of rTMS over control in improving manual dexterity, either immediately post-treatment (MD: 0.75; 95% CI: -4.62 to 6.11; *P* = 0.79) or in change-from-baseline comparisons (MD: 0.94; 95% CI: -4.71 to 6.59; *P* = 0.74). It aligns with the nuance seen in broader investigations: for instance, Chen et al.’s meta-analysis did identify a modest positive effect of rTMS on fine motor recovery (SMD = 0.38), though their inclusion criteria encompassed a variety of measures, not the BBT exclusively [20]. Meanwhile, more recent trials that targeted stimulation protocols or focused on specific stroke populations, such as cortical versus subcortical lesions, suggest that any measurable improvements in BBT may hinge on tailoring frequency, site, and timing of stimulation [21].

Null findings in grip strength are consistent with earlier literature; several studies that applied rTMS in isolation (without concomitant rehabilitative training) similarly failed to produce measurable gains in grip or pinch strength in stroke survivors [22]. Nonetheless, emerging meta-analyses point to a more nuanced narrative: excitatory rTMS, particularly high-frequency stimulation applied to the ipsilesional hemisphere, has demonstrated modest but significant improvements in hand strength, especially when paired with motor training and delivered within the first three months post-stroke [23].

The literature suggests that rTMS, when applied in isolation and without supplementary task-specific rehabilitation, rarely yields meaningful gains in fine motor control such as pinch strength. Indeed, the cortical neurophysiological markers, such as short-interval intracortical inhibition, correlate with pinch strength, but modulation through rTMS alone may be insufficient to translate into functional improvements [24]. Conversely, meta-analyses have shown that integrating rTMS with conventional rehabilitation, notably task-oriented training, is more likely to produce clinically relevant benefits in upper limb function, including pinch-related tasks, especially when stimulation is initiated early post-stroke [25].

Recent meta-analyses by Chen et al. and Tang et al. show that rTMS not only improves global upper-limb scores but also yields measurable gains in hand strength and fine motor dexterity, supporting the use of distal, task-based outcomes in stroke trials [20,23]. Consistent with our results, randomized studies applying low-frequency rTMS over the contralesional M1 followed by intensive hand practice report improvements in Action Research Arm test and Box and Block test performance, with gains closely linked to changes in motor-evoked potentials and interhemispheric balance [26].

Limitations and future research

This review has several important limitations that warrant acknowledgment. First, the number of eligible randomized controlled trials was small, restricting the statistical power and generalizability of the findings. Second, the included studies demonstrated heterogeneity in stimulation parameters, outcome measures, and follow-up durations, which complicates direct comparisons and may obscure subtle treatment effects. Third, most trials enrolled relatively small cohorts, often within single centers, thereby increasing susceptibility to bias and limiting external validity. Fourth, the absence of long-term follow-up data prevents firm conclusions regarding the durability of TMS-related changes. Finally, although efforts were made to minimize selection and reporting bias, the possibility of unpublished negative studies cannot be excluded.

Future research should move beyond broad assessments of upper limb recovery and focus explicitly on dexterity and fine hand function. Large, multicenter trials are required to enhance statistical robustness and generalizability. Stratifying patients by lesion location, stroke chronicity, and baseline impairment will help identify subgroups most likely to benefit. Protocols should also explore precision targeting of the motor hand area, optimal stimulation frequencies, and combinations with task-specific therapies such as robotics, virtual reality, or constraint-induced movement training. The integration of neuroimaging and neurophysiological biomarkers could further elucidate mechanisms of recovery, allowing for more personalized interventions. Finally, advancing TMS research with methodological rigor and clinical precision will be essential to unlocking its potential in restoring fine motor independence after stroke.

## Conclusions

This review found no significant evidence that TMS improves fine motor recovery after stroke. Current protocols may be insufficient to address the complexity of dexterity and hand function. Future research should focus on targeted stimulation strategies, larger trials, and integration with task-specific therapies to optimize outcomes.
